# Parental Perceptions and Practices toward Childhood Asthma

**DOI:** 10.1155/2016/6364194

**Published:** 2016-10-23

**Authors:** Amani K. Abu-Shaheen, Abdullah Nofal, Humariya Heena

**Affiliations:** ^1^Research Center, King Fahad Medical City, Riyadh, Saudi Arabia; ^2^Department of Emergency Medicine, King Saud University Medical City, Riyadh, Saudi Arabia

## Abstract

*Introduction*. Parental perceptions and practices are important for improving the asthma outcomes in children; indeed, evidence shows that parents of asthmatic children harbor considerable misperceptions of the disease.* Objective.* To investigate the perceptions and practices of parents toward asthma and its management in Saudi children.* Methods*. Using a self-administered questionnaire, a two-stage cross-sectional survey of parents of children aged between 3 and 15 years, was conducted from schools located in Riyadh province in central Saudi Arabia.* Results*. During the study interval, 2000 parents were asked to participate in the study; 1450 parents responded, of whom 600 (41.4%) reported that their children had asthma, dyspnea, or chest allergy (recurrent wheezing or coughing), while 478 (32.9%) of the parents reported that their children were diagnosed earlier with asthma by a physician. Therefore, the final statistical analyses were performed with 600 participants. Furthermore, 321 (53.5%) respondents believed that asthma is solely a hereditary disease. Interestingly, 361 (60.3%) were concerned about side effects of inhaled corticosteroids and 192 (32%) about the development of dependency on asthma medications. Almost 76% of parents had previously visited a pediatric emergency department during an asthma attack.* Conclusions*. Parents had misperceptions regarding asthma and exhibited ineffective practices in its management. Therefore, improving asthma care and compliance requires added parental education.

## 1. Introduction 

Asthma is a common chronic disease and remains an important public health issue, especially among the pediatric population [1–4]. Childhood asthma represents a significant burden, not only in terms of morbidity and reduced quality of life but also in terms of healthcare costs, as reflected by the high rates of unscheduled emergency department visits, hospitalization, and school absenteeism [5]. In developing countries, the terms “chest allergy” and “dyspnea” are commonly used instead of asthma to avoid the social stigma associated with the chronic nature of the disease [6].

The prevalence of childhood asthma is increasing rapidly, although the underlying reasons remain largely unknown. In Saudi Arabia, asthma has emerged as one of the most common chronic illnesses [7] mostly attributed to lifestyle changes owing to the modernization of Saudi society, the accompanying changes in dietary habits, and increasing exposure to environmental factors such as indoor allergens, dust, sand storms, and tobacco [8].

Parents are deemed to be the best judges of the severity of asthma because they can identify the symptoms as well as recognize their child's particular asthma pattern by careful, frequent observation [9]. Moreover, they are also familiar with the triggering factors, such as tobacco smoke and diet. Parental education is important for adherence to a treatment regimen. It is important to highlight the role of medications in asthma control and management because asthmatic children and their parents must understand and cooperate with new approaches to asthma care and control [10].

Parental perceptions and practices are crucial for improving the asthma outcomes in children; indeed, evidence shows that parents of asthmatic children harbor considerable misperceptions of the disease, and they often do not relate medication use to the prevention of asthma attacks [6]. The most striking findings supporting this notion were from the Asthma Insights and Reality in the Gulf and the Near East Survey, which found that parents tended to underestimate the severity of their child's asthma and overestimate the degree of asthma control [11, 12]. Moreover, there is a dearth of studies on parental perceptions and practices toward asthma reported in Saudi Arabia; hence, further research is required to increase awareness among healthcare providers to understand the effects of parents' perceptions and practices of asthma management. Thus, the aim of this study was to investigate the perceptions and practices of parents toward asthma and its management in Saudi children.

## 2. Materials and Methods

### 2.1. Overview

A two-stage cross-sectional survey was conducted of parents of children aged between 3 and 15 years from schools located in all five districts of Riyadh province located in central Saudi Arabia. The participants included in the survey represented a wide range of socioeconomic and demographic characteristics.

### 2.2. Recruitment of Schools

A list of all government and private primary schools in Riyadh was obtained from the General Directorate of Education in the Riyadh region (both boys' and girls' departments). Schools were stratified according to the geographical area, and five schools in each region were selected randomly.

### 2.3. Recruitment of Children

All enrolled students aged between 3 and 15 years in the selected schools were eligible to participate. A letter explaining the rationale for the study and what it entailed, consent forms, and questionnaires were sent to 2000 parents over a 5-month period in order to fulfill our required sample size of 1432.

### 2.4. Data Collection

Data collection was accomplished using a self-administered questionnaire which was based on the available literature to investigate the perceptions and the practices of parents of asthmatic children toward asthma and its management [6]. Data collection was performed in two phases: during the first phase, parents were asked whether the child suffered from asthma, chest allergy (a child with recurrent coughing especially at night, wheezing, trouble breathing or fast breathing, and frequent colds that settle in the chest), and/or dyspnea; then the participants were asked if their children have been previously diagnosed with asthma by a physician. The second stage of the questionnaire was specifically directed at parents of a child with asthma, chest allergy, or recurrent dyspnea.

The questionnaire was developed in the Arabic language and tested on 40 participants to ensure clarity and was updated according to their comments. The final questionnaire consisted of four sections. The first section of the questionnaire gathered demographic data, including age, gender, area of residence, educational level, number of children in the family, family income and history of asthma, consequences of suffering from asthma attack (absenteeism from school (once/month), difficulty in sleeping at night (once/month), and hospitalization), and symptoms of uncontrolled asthma. The other two sections assessed the perceptions and practices of parents of asthmatic children toward asthma and its management. We explored respondents' perceptions of the following: diagnosis, etiology of asthma, trigger factors, and medication side effects and dependency. We also identified respondents' practices regarding the following: preventive measures to reduce the likelihood of recurrence of asthma attacks (such as avoiding certain foods and strong smells), asthma attack management and ability to treat the child at home, regular visits to a physician (every 1–3 months), and whether they had previously visited a pediatric emergency department during an asthma attack. The final section enquired about the medications used in the treatment of asthma. The use of medications was determined by asking parents to list the current prescription asthma medications they had at home. The flow of this questionnaire allowed a smooth transition from primary to core study questions. Most questions were in a multiple-choice format and the participants were able to choose more than one answer to the same question.

### 2.5. Sample Size Calculation

Based on the literature the prevalence of asthma in Saudi Arabia is 23% [13]. Sample size for the current study is *n* = [*zα*/2*p∗q*/(*d∗d*)].

*p* = estimated prevalence = 0.23, *q* = 1 − *p* = 0.77, *d* = allowable error in estimating prevalence (margin of error) = 10% on either side of stated prevalence of 23% = 0.023, otherwise power of study = 90%, *α* = probability of type I error = 0.05 (2-sided), *z* = 1.96, therefore *n* = 1286.

Nonresponse rate = 10%, so the number of respondents needed for the study was 1432.

### 2.6. Ethical Considerations

Ethical approval was obtained from the Institutional Review Board at KFMC. Participants who met the inclusion criteria provided written informed consent.

### 2.7. Statistical Analysis

Data were analyzed using SPSS version 21.0 (SPSS, Inc., Chicago, IL, USA). Descriptive statistics (mean, standard deviation, and percentages) were used to describe the quantitative and categorical variables. Pearson's chi-squared test was used to compare the distribution of categorical variables. A *p* value less than 0.05 was considered to indicate significance.

## 3. Results

During the study interval, 1450 parents were recruited, of whom 600 (41.4%) reported that their children had asthma, dyspnea, or chest allergy (recurrent wheezing or coughing), while 478 (32.9%) of the parents reported that their children were diagnosed earlier with asthma by a physician. Therefore, the final statistical analysis was performed with 600 participants.

Three hundred seventy-two (62%) of the asthmatic children were males, with a mean age 7.22 (±3.6). Considering the area of residency, 80% of the included patients were from an urban residential setting, and 331 (55.2%) had a family history of asthma (Table 1).

Three hundred (50%) parents reported nocturnal dyspnea, as the most common symptom of uncontrolled asthma in children, whereas 31.3% observed a repetitive cough pattern. A total of 479 (79.8%) parents reported that their children had been previously hospitalized due to an asthma attack (Table 2).

The majority, 354 (82.7%), of asthmatic children were using a *β*-agonist as medications; only 6 (1.4%) children were reported using antihistamines (Table 3).

Table 4 shows the parent's perceptions toward asthma. When we asked the parents whether the child suffered from asthma, chest allergy, or recurrent dyspnea, 64.8% of the parents believed it was a chest allergy, 47.3% dyspnea, and 248 (41.3%) asthma. The majority of parents, 321 (53.5%), believed that asthma was a hereditary disease, and 462 (77.0%) cited exposure to dust as the potential trigger. This table also reveals the concern of parents about medication and its dependency. Of these parents, 361 (60.3%) were concerned about side effects of inhaled steroids and 192 (32%) about the development of dependency on asthma medications.

A total of 366 (61%) participants used vapor to treat their children. Moreover, 489 (81.5%) of parents avoided strong odors as a precaution. Almost 76% of parents had previously visited a pediatric emergency department during an asthma attack, whereas 323 (53.9%) regularly visited a physician (Table 5).

Table 6 presents the tabulation of the factors and parental concerns regarding the use of inhaled therapy in children with asthma. Family income and number of children in the family were significantly associated with parental concern about dependency (*p* ≤ 0.001 and 0.048, resp.). Parent education level was significantly associated with parental concern regarding side effects of inhaled steroids (*p* = 0.038). No statistically significant association was found between parental practices and demographic variables.

## 4. Discussion

This study provides valuable insight and critical cognizance about perceptions and various practices adopted by Saudi parents in asthma management. The diagnosis of asthma is based on clinical assessment as there is no gold standard diagnostic test. Asthma diagnosis in children should be based on a careful clinical assessment that includes recurrent or chronic symptoms related to airway obstruction, such as wheezing, coughing, night symptoms, activity limitation, and shortness of breath. The diagnosis can be further supported by the presence of atopy, early sensitization, and a family history of atopy [14–16]. Our results revealed that 600 (41.4%) participants reported that their children had asthma, dyspnea, or chest allergy (recurrent wheezing or coughing). Though, 478 (32.9%) parents of the total surveys reported that their children were earlier diagnosed for asthma by a physician. The significance of this study is that 600 (41.4%) respondents who were surveyed revealed about the presence of asthma, dyspnea, or chest allergy (recurrent wheezing or coughing) in their children, which may indicate that some cases may be undiagnosed or that they did not seek medical advice.

Our results revealed that the majority of parents believed that their child had chest allergy or dyspnea and did not identify asthma by name, which showed that the perceptions of participants were adversely affected by social stigma [6]. This fact is corroborated by a study reporting that parents preferred to hide the asthma diagnosis of their children from extended family members, friends, or the child's teachers due to the stigma of asthma. Also, the diagnosis of asthma is often denied by both children and their parents, so there is a need to anticipate the expectations in these families due to stigmatization. In fact, the terms chest allergy or recurrent dyspnea are preferred over asthma to mask the chronic nature and functional impairment of the illness [6, 17]. Another important observation was that only 53.9% of parents regularly visited pediatric clinics, whereas 75.8% of parents reported that they have visited pediatric emergency department previously during their child's asthma attack. Increased pediatric emergency hospitalization in Saudi Arabia is the result of improper medical awareness and utilization of services, which consequently causes an excessive economic burden on healthcare costs. Consistent with other studies [18–20], our results showed a high rate of hospitalization, difficulty in sleeping at night, and school absence due to asthma. Moreover, 50% of the parents reported nocturnal dyspnea. These findings indicate that asthma control and management in Saudi Arabia require proper guidelines and better awareness among parents. Besides, the findings reflected a lack of parental education regarding the role of medications in asthma management.

Moreover, the findings of this study on parental perceptions of asthma in a Saudi population revealed that 53.5% of parents of asthmatic children believed that asthma is solely a hereditary disease and interestingly 77% reported the dust or allergen a potent trigger factor. In support to our results, other studies conducted in the USA and India indicated that asthma was perceived as a hereditary disease [21, 22], whereas a study from Pakistan showed that it was viewed largely as a contagious disease [1]. The discrepancies between populations in the perception of asthma etiology are probably related to the educational level, the quality of information provided by the treating physicians, and the structure of the health care system. To have better treatment outcomes, it is important to explain to the parents that asthma is not solely hereditary, but environmental factors play a major role in susceptibility and triggering of attacks.

In order to enhance the level of perceptions among asthmatic patients and caregivers, education should include knowledge about asthma and its management, as there might be misperceptions about the use of inhalers and the safety of inhaled corticosteroids (ICS). Parents often express concern about the safety of asthma medications. Chan and DeBruyne [23] reported that high proportions of parents were concerned about the side effects of asthma medications (91%) and inhaler dependency (86%). Furthermore, parental concern regarding medication dependence was reported as a reason for weaning children off the drugs [24]. In our study, the majority 361 (60.3%) of parents with asthmatic children were concerned about the side effects of ICS, and 192 (32%) parents were concerned about dependency on medications, which may be related to lack of knowledge about asthma, lack of partnership in the management, inappropriate expectations, and cultural issues. It is widely known that improving adherence to inhaled corticosteroids (ICS) in children with asthma is probably the most effective method through which healthcare providers can reduce the burden of uncontrolled asthma [21]. However, unfortunately the majority of parents with asthmatic children from Asian countries view ICS administration to their children as a means of becoming addicted to or dependent on inhaler use, a misconception possibly related to modern day inhalational drug abuse and steroid abuse driven by extensive media campaigns [22]. These concerns may likely prompt the children to miss the prescribed doses of inhaled steroids with increased problems of nonadherence to treatment as well as higher number of consequent hospitalizations. In contrast, parents from the western countries based the ICS maintenance treatment of the children as per the need of their child's health, convenience of administration, and the level of harm from medicines.

The results revealed interesting findings of parental practices towards asthmatic children in Saudi Arabia, where about 82.7% patients use *β*-agonist, while only 7.2% use inhaled steroids, which does not meet the guidelines of Saudi Initiative for Asthma (SINA) with regard to the usage of inhaled corticosteroids ICS as the most effective anti-inflammatory medications for the treatment of asthma. SINA was developed in 2009 with special attention directed towards nonasthma specialists, including primary care and general practice physicians. SINA guidelines were initially based on two existing international guidelines, the Global Initiative for Asthma (GINA) and the National Asthma Education and Prevention Program [14–16, 25–27]. A study by Al-Kabbaa et al. found that 39% of primary care physicians meet the standards of the national guidelines in management of asthma. However, the overall level of awareness among physicians was low (52%). Their proficiency in general knowledge, diagnosis, classification of severity, and management was also low [28].

Regarding parental practices toward asthma control, 81.5% of parents avoided strong odors, whereas 61% of parents use vapor treatment for asthma control and management.

Moreover, parents with lower family incomes and a higher number of children were concerned more about medication dependency and parents with lower education levels were more concerned about the side effects of inhaled steroids compared with more highly educated parents. This finding can be considered as lack of awareness and underutilization of medical facilities in management and control of asthma which might also explain an increased asthma prevalence among lower socioeconomic groups with education also being a determinant of asthma risk [29]. Parental education is crucial for successful treatment of this disease, and it is important that the treating physician emphasizes the role of effective medications in asthma control. It is necessary to inform parents/children about the long-term course of asthma and point out the safety of prolonged inhaled steroid use. Thus, health care workers should be aware about the prevalent beliefs and misconceptions among the parents about asthma control and management [29, 30].

The overall prevalence of asthma in Saudi children has been reported to be 8–25% in studies conducted over the past three decades. The highest prevalence of physician-diagnosed asthma in Saudi Arabia was 25% in 2004 [31]. Epidemiological studies carried out in Saudi Arabia have revealed that the prevalence of asthma has considerably increased which may be attributed to rapid lifestyle changes related to the modernization of Saudi society, changes in dietary habits, and increased exposure to environmental factors such as indoor allergens, dust, sandstorms, and tobacco [32, 33]. Other possibilities include the hygiene hypothesis, which proposes insufficient exposure to microbes early in life due to pharmacological manipulations and vaccines [9]. In recent decades, very few studies have been found in our databases highlighting the parental perceptions and practices towards asthma management. To understand the present lacuna in asthma control and management, the health care system and parental education are the key component, which needs to be improved in order to better control asthma. The government of Saudi Arabia has given high priority to the development of health care services at all levels: primary, secondary, and tertiary. As a consequence, the health of the Saudi population has greatly improved in recent decades. However, a number of issues pose challenges to the health care system, such as a shortage of Saudi health professionals, effective partnership between patients and their healthcare providers, changing patterns of disease, high demand resulting from free healthcare services for all citizens and those expatriates working within the public sector, and poor accessibility to some health care facilities; therefore many people experience long waiting lists for various health care services and facilities [34]. Additionally, there is a dearth of services for disadvantaged groups particularly those in rural areas [35]. Therefore, it is crucial to have accurate and updated health information for planning. Moreover, focusing on parental education can improve the management of asthmatic children [36, 37] because improved parental education may not only lead to better practices in asthma control and management among children but also help in proper medication utilization. Education aimed at parents should be targeted to change these perceptions and practices among parents of asthmatic children in Saudi Arabia. To avoid further health challenges, asthma needs to be diagnosed in its early childhood stages, which in turn requires effective parental knowledge and practices. The parents should be guided by the health care worker that asthma is a long-term condition requiring long-term care and the knowledge about the causes of symptoms and the warning signs requiring emergency care should be clearly imparted to them. Also, the known triggers and their avoidance, the role of inhalers, and their proper use need to be stressed including regular use for prevention of acute attacks. The strength of our study is that exploration of parents' perceptions and practices toward asthma and its management would provide evidence-based knowledge, which will not only help in improving health care but also provide a basis for construction of guidance manuals for parents and medical staff. The self-evaluated questionnaire highlighting the sociodemographic parameters, with questions related to perception about disease inheritance, etiology, and precautions, can serve as a useful tool to explore the overlooked areas in this research arena. The critical evaluation of questionnaire will aid the researchers and health care providers in drafting guidance manuals and policies to combat the further prevalence of this life long illness, whereas one of the limitations of our study is that we were not able to confirm the prescribed medication or the diagnosis by medical record review. Moreover, regarding the magnitude of asthma/chest allergy in this study, a limitation is that there is no nonrespondent analysis.

## 5. Conclusions

The present study provides a valuable understanding of asthma perceptions and practices in Saudi Arabia, which have an important role in the explanation of distress outcomes across a range of respiratory health conditions. It may be a harsh admission that prevailing misperceptions in asthma are directly responsible for inefficient and inadequate practices taken for asthma control. To improve asthma care and compliance, the medical community should take initiatives to provide proper education and guidelines and also conduct awareness events, for example, workshops to break social stigma toward asthma. This preliminary data highlights the causes which usually remain unnoticed, but which play a pivotal role in the management of illness. Further longitudinal nationwide studies at the community level encompassing different regions of the kingdom of Saudi Arabia are needed to fully assess the magnitude as well as provide a base for the development of general guidelines and preventive programs for health education. After having said that, the quality of the existing health education and health promotion has to be improved by bringing such issues to the forefront for the stake holders and decision makers.

## Figures and Tables

**Table 1:** Sociodemographic characteristics of asthmatic children and their parents (*n* = 600).

Characteristics	Number (%)
*Age (years)*	
3–5	208 (34.7)
6–8	152 (25.3)
9–11	124 (20.7)
≥12	116 (19.3)
*Gender*	
Male	372 (62.0)
Female	228 (38.0)
*Area of residency*	
Urban	480 (80)
Rural	120 (20)
*Parent education level*	
Illiterate	59 (9.8)
Secondary	253 (42.2)
University	235 (39.2)
Higher education	53 (8.8)
*Family income (month)*	
<10,000 (low)	317 (52.8)
10,00–20,000 (middle)	192 (32)
>20,000 (high)	91 (15.2)
*Number of children in the family*	
1–5	307 (51.2)
6–10	260 (43.3)
>10	33 (5.5)
*Family history of asthma *	331 (55.2)

**Table 2:** Consequence and symptoms of uncontrolled asthma.

	Number (%)
*Consequence of asthma*	
Hospitalization	479 (79.8)
Difficulty in sleeping at night (once/month)	350 (58.3)
School absenteeism (once/month)	312 (52)
*Symptoms of uncontrolled asthma*	
Nocturnal dyspnea (more than one night/week)	300 (50.0)
Exercise dyspnea	239 (39.8)
Dyspnea at rest	222 (37.0)
Repetitive cough (daily)	188 (31.3)

**Table 3:** Asthma medications used for participated children (*n* = 429).

Medication	Number (%)
Inhaled *β*-agonist	354 (82.7)
Inhaled corticosteroids	31 (7.2)
O_2_	20 (4.6)
Leukotriene antagonists	11 (2.5)
Oral steroids	7 (1.6)
Antihistamines	6 (1.4)

**Table 4:** Perceptions toward asthma by parents of asthmatic children.

Parental beliefs	Total number of participants	Percentage (%)
Chest allergy (recurrent wheezing or coughing)	389	(64.8)
Dyspnea	284	(47.3)
Asthma	248	(41.3)
*Etiology*		
Hereditary	321	(53.5)
Acquired	82	(13.7)
Others	197	(32.8)
*Triggers*		
Dust	462	(77.0)
Indoor smoking	219	(36.5)
Virus	177	(29.5)
Food	86	(14.3)
*Worries about medications*		
Inhaled steroid side effects	361	(60.3)
Inhaler side effects	320	(53.3)
Dependency	192	(32.0)

**Table 5:** Practices toward asthma by parents of asthmatic children.

Parental practices	Total number of participants	Percentage (%)
*Ability to treat at home*	367	(61.2)
*Treatment used*		
Vapor	366	(61.0)
Medicine	238	(39.7)
Massage	142	(23.7)
Herb	102	(17)
Certain foods	25	(4.2)
*Precautions used*		
Avoiding strong odor	489	(81.5)
Avoiding indoor smoking	482	(80.3)
Regular use of medicines	454	(75.7)
Avoiding cold weather	446	(74.3)
Avoiding physical exercise	346	(57.7)
Avoiding certain foods	326	(54.3)
*Visiting pediatric emergency *	455	(75.8)
*Regular visit to physician *	323	(53.9)

**Table 6:** Factors and parental perception that influence concerns towards the use of inhaled therapy in children with asthma.

Variables	Worries from dependency *n* (%)	*p* value
*Family income*		
Low	125 (39.4)	≤0.001
Middle	49 (25.5)	
High	18 (19.8)	
*Number of children in the family*		
1–5	86 (28.0)	0.048
6–10	91 (35.0)	
>10	15 (45.4)	

	Worries from inhaler steroids side effects	

*Parent education level*		
Illiterate	39 (66.1)	0.038
Secondary	159 (62.8)	
University	138 (58.7)	
Higher education	25 (47.2)	
